# Supplementary material for: Application of Aptamers Improves CRISPR-Based Live Imaging of Plant Telomeres
Source: Front Plant Sci. 2020 Aug 20;11:1254. doi: 10.3389/fpls.2020.01254 (PMC7468473; doi:10.3389/fpls.2020.01254)
Supplement: Supplementary file 1 [file DataSheet_1.pdf]

Supplementary Table 1. List of primers used in cloning steps and PCR reactions.

**Primer name**	**Primer sequence (5' to 3')**
Apta2-FWD	AAGTGGCACCGAGTCGGTGCTTTTTTTCTAGACCCAGCTTTCTTGTACa
Apta2-Rev	accggtGTACAAGAAAGCTGGGTCTAGAAAAAAAGCACCGACTCGGTGCCACTT
Apta2-Rev2	CCGACTCGGTGCCACTTCC
SS42	CGACTAAGGGTTTCTTATATGC
ecori-35s-f1	AAGCAGAATTCAACATGGTGGAGCACGACACA
ecori-35s-r1	TGCTTAGCGAATTCCCCCCGTGTTCTCTCCAAATG
PRS5A-FWD	gaattcGATGAGAGAGGAACTG
PRS5A-REV	gaattcGGTGAGAGAAACAGAG
Cas9-XVE-F	acagctatgacatgattacgaattcATAGTTTAAACTGAAGGCGGGAAAC
XVE-Lexa-A-R	atcaattcccTCAGACTGTGGCAGGGAAAC
XVE-Lexa-A-F	cacagtctgaGGGAATTGATCCCCCCTC
LexA-Cas9-R	ttcttatccatggcgcgccgaatTCCTCTCCAAATGAAATGAACTTC
MS2(NLS)-GFP#1 f	aagagaaaggttgcagctgctATGGTGAGCAAGGGCGAG
GFP#1-linker1-r	atgccagagcggccgccagaCTTGTACAGCTCGTCCATG
linker1-GFP#2-f	agtctggcggccgctctggcATGGTGAGCAAGGGCGAG
GFP#2-linker2-r	atgctaccatcgatgctaccCTTGTACAGCTCGTCCATG

linker2-GFP#3-f	agggtagcatcgatggtagcATGGTGAGCAAGGGCGAG
GFP#3-nos_ter-r	ggggaaattcgggggcaattTTACTTGTACAGCTCGTCC
MS2(NLS)-mRuby#1-f	aagagaaaggttgcagctgctATGGTGTCTAAGGGCGAAG
mRuby#1-linker1-r	atgccagagcggccgccagaCTTGTACAGCTCGTCCATC
linker1-mRuby#2-f	agtctggcggccgctctggcATGGTGTCTAAGGGCGAAG
mRuby#2-linker2-r	atgctaccatcgatgctaccCTTGTACAGCTCGTCCATC
linker2-mRuby#3-f	agggtagcatcgatggtagcATGGTGTCTAAGGGCGAAG
mRuby#3-nos_ter-r	ggggaaattcgggggcaattTTACTTGTACAGCTCGTCCA
pDS2.0-ΔsgRNA-r	aGAGACGTCCGTCTCcCAAT
pDS2.0-ΔsgRNA-f	AAGTGGCACCGAGTCGGTG
sgRNA2.0-MS2-flip/ext-f	attgggagacggacgtctctGTTTAAGAGCTATGCTGGGCCAAC
sgRNA2.0-MS2-flip/ext-r	attgggagacggacgtctctGTTTAAGAGCTATGCTGGGAGCAG
Telomere protospacer-F	attgGGGTTTAGGGTTTAGGGTTT
Telomere protospacer-R	aaacAAACCCTAAACCCTAAACCC
Centromere protospacer1-F	attgACCTTCTTCTTGCTTCTCAA
Centromere protospacer1-R	aaacTTGAGAAGCAAGAAGAAGGT
Centromere protospacer2-F	attgTCTTCTTGCTTCTCAAAGCT
Centromere protospacer2-R	aaacAGCTTTGAGAAGCAAGAAGA
Centromere protospacer3-F	attgATATGAGTCTTTGGCTTTGT
Centromere protospacer1-R	aaacACAAAGCCAAAGACTCATAT
GFP-F	CCGACAAGCAGAAGAACGGC

GFP-R	GCGCTTCTCGTTGGGGTCTT
dCas-F	AGGTGGCATACCACGAGAAG
dCas-R	TGGTTGTAGGTCTGCACGAG

Supplementary Table 2. Summary of CRISPR live-cell imaging in stably transformed plants.

**Species**	**Construct used for transformation**	**dCas9 promoter**	**Target region**	**No. of gentamycin resistant plants and root lines analyzed by microscopy**	**No. of plants with signals**	**Signal description**
N. benthamiana	dCas9:2xMS2:GFP	ubiquitin	telomere	30 plants	7	weak uniform labelling of nuclei
N. benthamiana	dCas9:2xMS2:GFP	CaMV 35S	telomere	33 plants	5	uniform labelling of nuclei
A. thaliana	dCas9:2xMS2:GFP	ubiquitin	centromere	141 plants	27	dot-like and uniform labeling of nuclei
A. thaliana	dCas9:2xMS2:GFP	ubiquitin	telomere	-	3	no signal or one signal only
A. thaliana	dCas9:2xMS2:GFP	CaMV 35S	telomere	18 plants	4	uniform labeling of leaf nuclei and stomata
A. thaliana	dCas9:2xMS2:GFP	XVE	centromere	7 plants	7	uniform labelling
A. thaliana	dCas9:2xMS2:GFP	XVE	telomere	6 plants	6	some nuclei with many dot-like signals
D. carota	dCas9:2xMS2:GFP	ubiquitin	telomere	15 root lines	4	weak uniform labelling of nuclei
D. carota	dCas9:2xMS2:GFP	CaMV 35S	telomere	12 root lines	1	weak uniform labelling of nuclei
