# Supplementary figures and images for: Application of Aptamers Improves CRISPR-Based Live Imaging of Plant Telomeres
Source: Front Plant Sci. 2020 Aug 20;11:1254. doi: 10.3389/fpls.2020.01254 (PMC7468473; doi:10.3389/fpls.2020.01254)

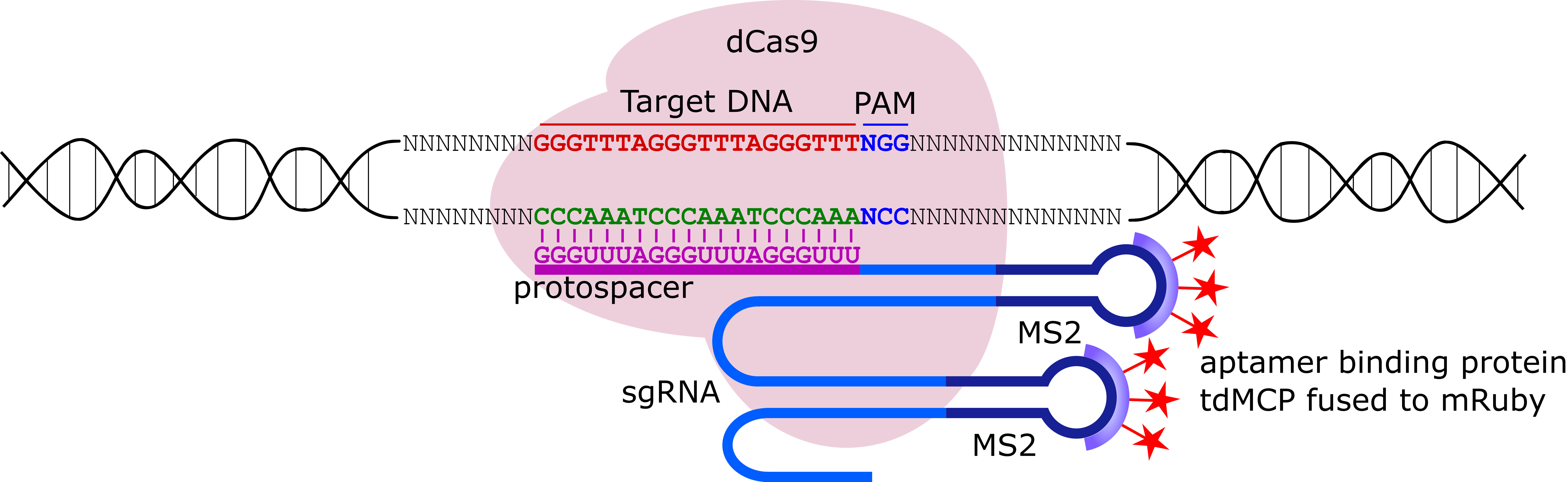

Supplement: Figure supplement 1 — Different components of the aptamer-based labeling method: 1) dCas9 from S. pyogenes, 2) MS2 or PP7 aptamers (here only MS2 is shown) which are integrated into sgRNA scaffold. 3) RNA binding protein (tdMCP or tdPCP) fused to a fluorescent protein (mRuby) which recognizes aptamers. [file Image_1.jpeg]

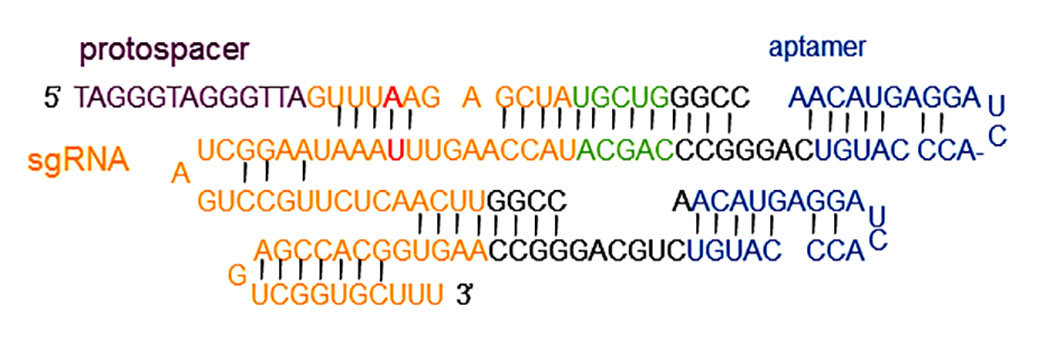

Supplement: Figure supplement 2 — Changing the sgRNA scaffold with A/U flip (in red) and insertion of an extension (in green). [file Image_2.jpeg]

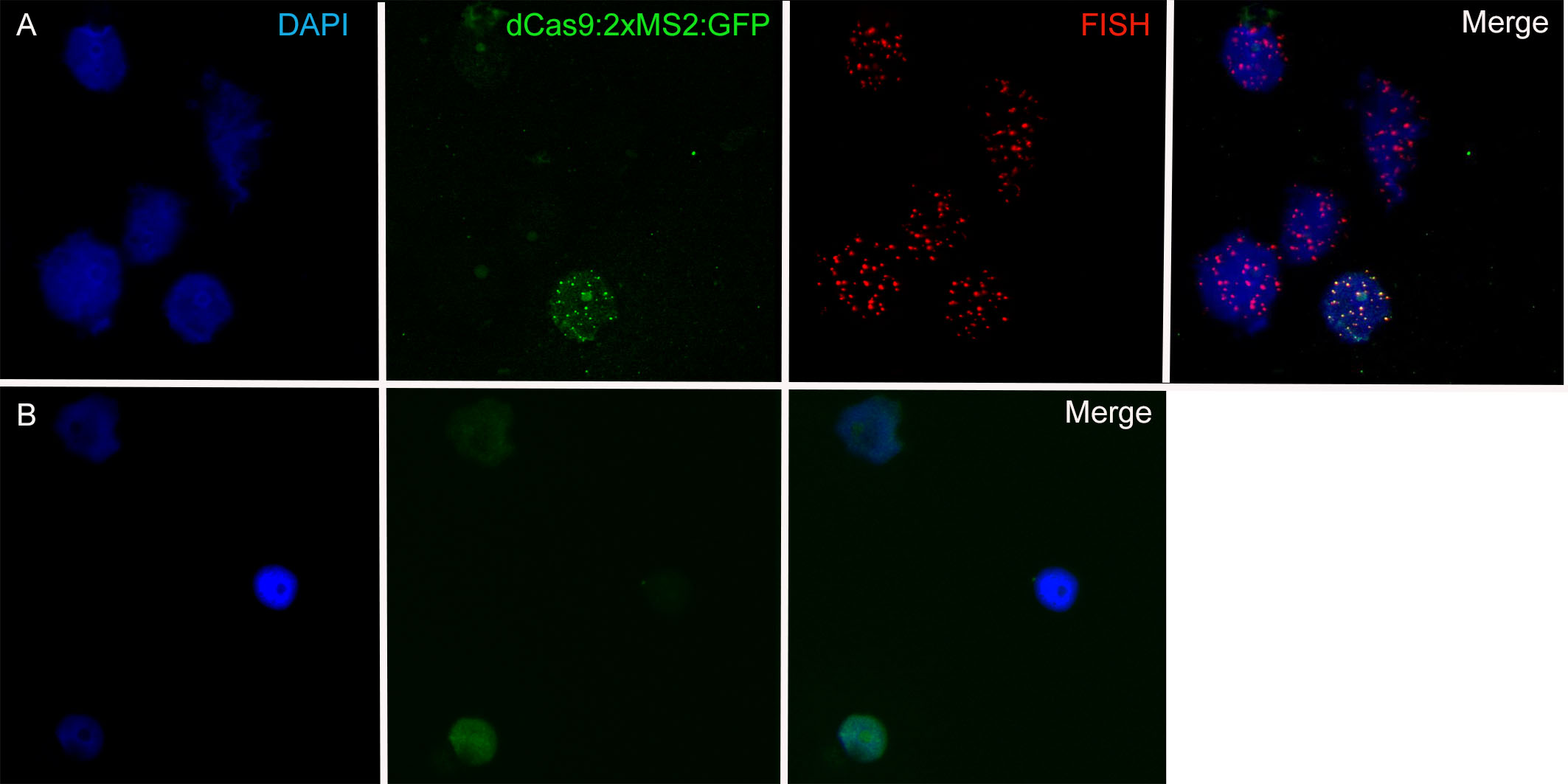

Supplement: Figure supplement 3 — Specificity control test by ImmunoFISH for the activity of the inducible XVE promoter. (A) Isolated nuclei after treatment of leaves with β-estradiol show telomeric signals. Co-localization of dCas9:2xMS2:GFP and FISH signals show that the observed signals are telomeric specific. (B) Nuclei isolated from β-estradiol-untreated leaves show a uniform labeling of nuclei. [file Image_3.jpeg]

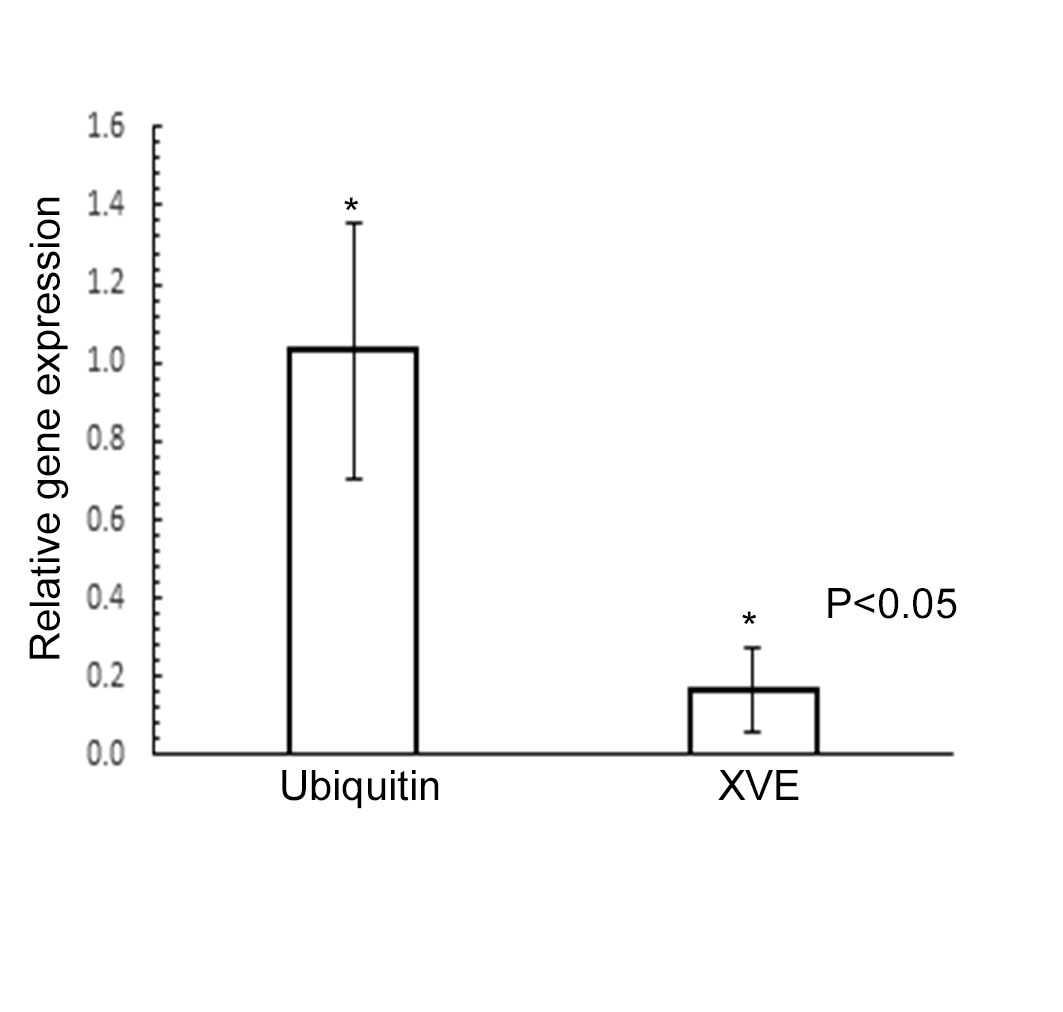

Supplement: Figure supplement 4 — Real time expression of dCas9 expressed by ubiquitin and XVE promoters. dCas9 expression is much lower when it is driven by inducible XVE promoter compared to ubiquitin from parsley. Error bars are standard deviation. [file Image_4.jpeg]

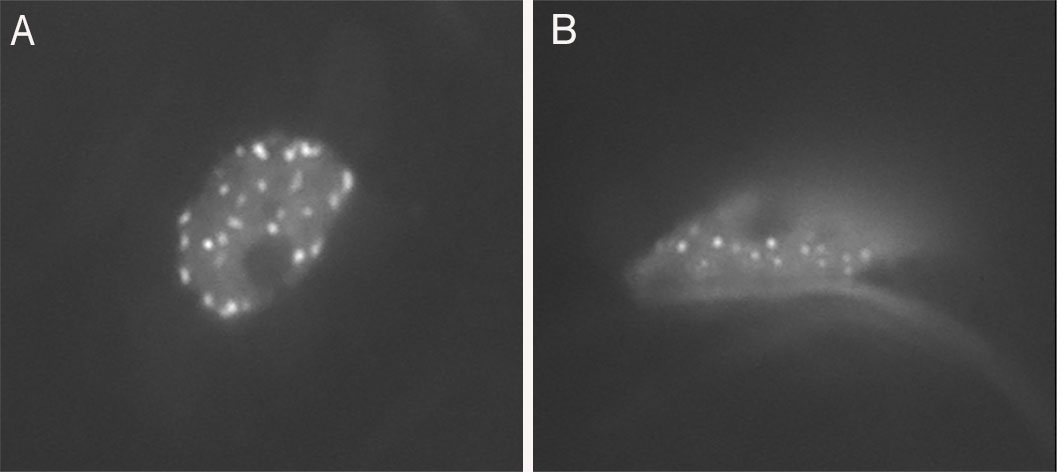

Supplement: Figure supplement 5 — Selected nuclei of A. thaliana stably transformed with a centromere-specific dCas9:2xMS2:GFP construct exhibiting dot-like signals. (A, B) Application of the centromere-specific protospacer 1 and 2, respectively. The number of signals was higher than expected. [file Image_5.jpeg]

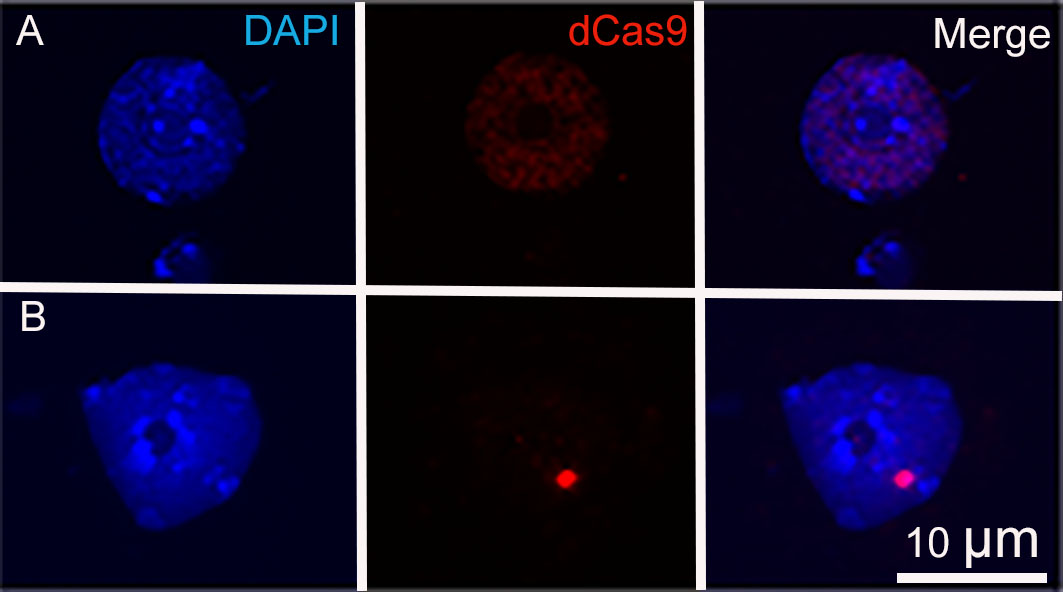

Supplement: Figure supplement 6 — Immunostaining of dCas9 protein in isolated nuclei from leaf material of stably transformed Arabidopsis plants with dCas9:2xMS2:GFP targeting centromeric regions. (A) Immunostaining of dCas9 in stably transformed Arabidopsis plants showed the dCas9 is not degraded. (B) Immunostaining of isolated leaf nuclei from wild type Arabidopsis leaf nuclei did not result in signals which shows that the applied antibody against dCas9 is working specifically. [file Image_6.jpeg]
